# From Orange to Oncology: Anti-Inflammatory and Anti-Cancer Mechanisms of Sinensetin

**DOI:** 10.3390/cells15020110

**Published:** 2026-01-08

**Authors:** Dong Joon Kim, Songyeon Ahn, Xiaomeng Xie, Yeon-Sun Seong, Yong Weon Yi

**Affiliations:** 1 Department of Microbiology, College of Medicine, Dankook University, Cheonan-si 31116, Chungcheongnam-do, Republic of Korea; 2 Multidrug-Resistant Refractory Cancer Convergence Research Center (MRCRC), Dankook University, Cheonan-si 31116, Chungcheongnam-do, Republic of Korea; 3 Department of Pathophysiology, School of Basic Medical Sciences, Academy of Medical Science, College of Medicine, Zhengzhou University, Zhengzhou 450008, China; 4 China-US (Henan) Hormel Cancer Institute, Zhengzhou 450008, China; 5 Department of Pharmacology, College of Medicine, Dankook University, Cheonan-si 31116, Chungcheongnam-do, Republic of Korea; 6 Chest Hospital of Zhengzhou University, Zhengzhou 450008, China; 7 Department of Biochemistry, College of Medicine, Dankook University, Cheonan-si 31116, Chungcheongnam-do, Republic of Korea

**Keywords:** anti-inflammatory, anticancer, immune response, sinensetin

## Abstract

**Highlights:**

**What are the main findings?**

- Sinensetin is a citrus-derived flavone that consistently shows anti-inflammatory activities across models by suppressing inflammatory signaling and supporting antioxidant/autophagy programs.
- Across multiple cancers, sinensetin inhibits tumor-relevant signaling (including MKK6/P38 signaling), alongside emerging immune-related effects.

**What is the implication of the main finding?**

- Multiple mechanisms support sinensetin as a multi-target lead for chemoprevention or adjunct therapy.

**Abstract:**

Sinensetin, a polymethoxylated flavone abundant in citrus fruits, has been recognized for its broad biological activities and wide use in traditional medicine around the world. Emerging clinical evidence from flavonoid-enriched orange juice interventions indicates antioxidant and anti-inflammatory effects, aligning with extensive preclinical data. In this review, we explored in vitro and in vivo findings on the anti-inflammatory and anticancer actions of sinensetin and delineated the underlying cellular pathways, especially in terms of proposed targets for sinensetin. In inflammatory settings, sinensetin attenuates NF-κB activation, lowers pro-inflammatory cytokines (e.g., TNF-α, IL-6), and enhances antioxidant defenses, supporting its reported antioxidant, anti-bacterial, anti-viral, and anti-obesity properties. Across multiple tumor models, sinensetin suppresses oncogenic signaling—including β-catenin, PI3K/AKT, VEGF, NRF2, P53, and MKK6—concomitant with reduced proliferation, migration, and survival signaling. We further discuss emerging immunological effects, including modulation of innate immune cell activation and cytokine production, which may contribute to tumor microenvironment reprogramming and inflammation resolution. Together, these mechanistic insights position sinensetin as a promising lead for chemopreventive and adjunct therapeutic strategies. Our efforts aim to provide insights into the future translational development and clinical evaluation of sinensetin and its derivatives.

## 1. Introduction

**Figure 1:**
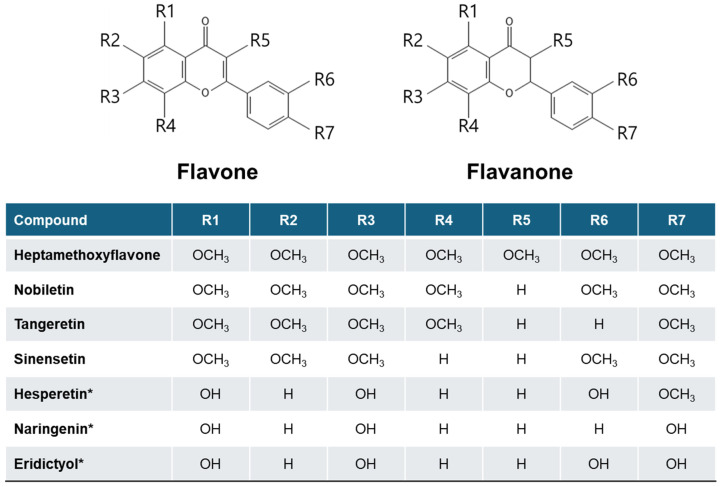
Flavonoids abundant in orange (Citrus fruit). Substitution pattern at positions R1–R7 on the common 2-phenylchromen-4-one (flavone) or 2-phenylchroman-4-one (* flavanone) backbone.

Natural products have been explored as sources of dietary supplements and pharmaceuticals throughout human history due to their structural diversity and wide range of biological activities [1]. *Orthosiphon aristatus (B1) Miq.* [synonyms: *O. stamineus* Benth, *O. grandifloras* Bold., *O. spicatus* (Thumb.) Bak.; Lamiaceae] [2] has been widely used in traditional medicine across various countries, including China and parts of Europe, to treat conditions such as cancer, hypertension, rheumatism, tonsillitis, gout, menstrual disorders, diabetes, and diuresis [3,4,5,6,7]. One of the active compounds isolated from *O. aristatus* is sinensetin (Figure 1) [7,8,9]. Sinensetin is a pentamethoxy-flavone that is also present in several citrus fruits [10].

Generally, the most abundant flavonoids in sweet orange (*Citrus sinensis*) fruit and juice are flavanone glycosides such as hesperidin. Flavanones are known to be beneficial for human health, primarily through antioxidant activities. However, polymethoxyflavones (PMFs) have gained more attention due to the increasing evidence supporting their activities, including antioxidant, anti-inflammatory, anticancer, regulation of metabolic syndrome, and immune system, etc. [11]. Among several PMFs, Lam et al. showed that sinensetin exhibits the best anti-angiogenesis effect compared to nobiletin, hesperetin, scutellarein, neohesperidin, and naringin in HUVECs and zebrafish models [12]. Furthermore, sinensetin exhibits a broad spectrum of pharmacological activities, including anticancer, antioxidant, and anti-inflammatory effects [7,13,14]. Moreover, sinensetin has been shown to sensitize cancer cells to chemotherapeutic drugs by downregulating or inhibiting multidrug resistance proteins such as P-glycoprotein and ABCG2 (ATP-binding cassette subfamily G member 2), although the precise mechanisms underlying these effects remain unclear [8,15,16,17,18]. In this review, we examine the cellular signaling pathways, summarize in vitro and in vivo evidence for sinensetin’s anti-inflammatory, anticancer, and immune cell activities, and propose molecular targets with particular emphasis on our recent work identifying selective inhibition of MMK6 (mitogen-activated protein kinase kinase 6) [19]. Together, these mechanistic insights position sinensetin as a promising lead for chemopreventive and adjuvant therapeutic strategies.

## 2. Flavonoids in Oranges

Citrus fruits—such as oranges, mandarins, tangerines, grapefruits, and lemons—contain diverse flavonoids concentrated in the peel (flavedo/albedo) and, to a lesser extent, the pulp. These compounds act as antioxidants and modulators of inflammation, metabolic enzymes, and cell-signaling pathways [20]. In sweet orange (*Citrus sinensis*), the dominant flavanones in the juice and pulp are hesperidin, narirutin, and didymin, while the peel is enriched in PMFs such as nobiletin, tangeretin, and sinensetin (Table 1); other flavonoids (e.g., diosmin and anthocyanins in blood oranges) differ by the variety.

Hesperidin (hesperetin) activates the NRF2 (nuclear factor erythroid 2-related factor 2)—ARE (antioxidant responsive element) axis, upregulating phase-II/antioxidant defenses [e.g., NQO1 (NAD(P)H:quinone oxidoreductase 1), SOD (superoxide dismutase), and CAT (catalase)] and lowering reactive oxygen species (ROS). It also exerts anti-apoptotic and pro-survival effects through balancing the PI3K (phosphoinositide 3-kinase)/AKT and MAPK (mitogen-activated protein kinase) signaling. Together these mechanisms support anti-inflammatory and cytoprotective actions [25,26]. Narirutin (naringenin) shows anti-inflammatory and antioxidant activity via NF-κB (nuclear factor κB-light-chain-enhancer of activated B cells) downregulation and NRF2 upregulation, alongside metabolic benefits linked to AMPK (AMP-activated protein kinase) activation and suppression of lipogenesis [e.g., SREBP1c (sterol regulatory element-binding protein 1c)], contributing to improved lipid handling and insulin sensitivity in preclinical and emerging clinical data [27,28]. Didymin (diosmetin) inhibits the PI3K/AKT/mTOR (mammalian target of rapamycin) pathway and promotes caspase-dependent apoptosis in tumor models, with additional anti-inflammatory signals reported. These effects align with diosmetin’s pro-apoptotic and autophagy-inducing actions in cancer contexts [29,30].

Among PMFs, nobiletin improves metabolic profiles by activating AMPK and modulating lipid/glucose pathways (e.g., SREBP and lipogenic enzymes) and also enhances circadian clock output [e.g., CLOCK (circadian locomotor output cycles protein kaput)–BMAL1 (basic helix-loop-helix ARNT like 1)/ROR (receptor tyrosine kinase-like orphan receptor) axis], which is linked to anti-obesity and insulin-sensitizing effects in models [31,32]. Tangeretin attenuates the PI3K/AKT and ERK (extracellular signal-regulated kinase) signaling, induces cell-cycle arrest, and activates caspases-3/8/9, supporting antiproliferative and chemosensitizing effects in preclinical systems [33,34]. Lastly, sinensetin exhibits anti-inflammatory actions and inhibits angiogenesis by targeting various kinds of signaling, which we will discuss further in the following sections.

## 3. Anti-Inflammatory Mechanism of Sinensetin

A randomized, double-blind, placebo-controlled trial in adults with obesity found that the group that received flavonoid-enriched orange juice (treatment group) showed increased total antioxidant capacity concomitant with upregulation of GPX1 (glutathione peroxidase-1), indicating enhanced systemic redox defense [35]. Additionally, circulating pro-inflammatory cytokines decreased in the treatment group, including TNFα (tumor necrosis factor-alpha) and IFNγ (interferon-gamma), consistent with an anti-inflammatory effect of dietary PMF [35]. Together, this study suggests that citrus flavonoids can be effective clinically. However, there was no specific mechanism for the anti-inflammatory effect suggested in this study. There are several preclinical studies investigating the anti-inflammatory mechanisms of sinensetin (Table 2).

### 3.1. AMPK Pathway

The potential protective effect of sinensetin on osteoarthritis (OA) has been reported. Sinensetin did not exhibit cytotoxic effects on the primary chondrocytes at concentrations up to 40 μM, but it demonstrated anti-apoptotic effects in tert-butyl hydroperoxide (TBHP)-treated cells [36]. Additionally, sinensetin negatively regulated TBHP-induced extracellular matrix (ECM) degradation and promoted autophagy in TBHP-treated chondrocytes [36]. Similar to its effects in mature adipocytes [44], sinensetin increased phospho (p)-AMPK levels while decreasing p-mTOR levels in a dose-dependent manner [36].

Furthermore, AMPK-dependent regulation by sinensetin has been reported in a rat model of colitis. In 2,4,6-trinitrobenzene sulfonic acid (TNBS)-induced colitis model rats, sinensetin reduced inflammatory responses and enhanced autophagy of epithelial cells [37]. Sinensetin administration restored epithelial barrier functions by promoting autophagy-mediated inhibition of apoptosis and preventing claudin-2 degradation [37]. These protective effects on barrier functions were suggested to be mediated by the AMPK/ULK1 (Unc-51-like kinase 1) pathway, as siRNA-mediated depletion of AMPK abolished epithelial autophagy in Caco-2 cells [37].

### 3.2. NF-κB Pathway

Regulation of the NF-κB pathway by sinensetin has been reported in neuronal cells. Deposition of Aβ (amyloid β) peptides is considered to be a major pathological driver of Alzheimer’s disease (AD) [45]. Extracellular accumulation of Aβ aggregates induces neuronal toxicity through oxidative stress, inflammation, and apoptosis [45]. In cultured neuronal cells, sinensetin inhibited activation of the TLR4 (Toll-like receptor 4)/NF-κB pathway induced by Aβ_25–35_ [38]. Moreover, sinensetin dose-dependently reduced Aβ_25–35_-induced cytotoxicity in SH-SY5Y cells, but it did not exhibit cytotoxicity to SH-SY5Y cells in normal conditions [38]. Sinensetin also upregulated the expression of antioxidant molecules such as CAT, GSH (glutathione), and SOD in Aβ-treated cells [38]. In addition, sinensetin downregulated Aβ-induced inflammatory mediators, including nitrite, COX2 (cyclooxygenase-2; also known as PTGS2), IL1β (interleukin-1β), iNOS (inducible nitric oxide synthase; also known as NOS2), and TNFα [38]. Mechanistically, sinensetin attenuated the Aβ-induced upregulation of TLR4 and nuclear translocation of NF-κB P65 [38]. Since overexpression of TLR4 abrogated the effects of sinensetin on the Aβ-mediated neurotoxicity, apoptosis, and downregulation of antioxidant molecules, TLR4 is suggested as the major target for the neuroprotective effects of sinensetin [38]. Furthermore, sinensetin has been reported to inhibit BACE1 (β-site amyloid precursor protein-cleaving enzyme 1) activity in vitro [39], and BACE1 upregulation has been linked to NF-κB-mediated oxidative stress and inflammation in AD [46]. However, the precise mechanisms by which sinensetin regulates the TLR4/NF-κB axis remain to be elucidated.

In a lipopolysaccharide (LPS)-induced acute pulmonary inflammation mouse model, sinensetin attenuated lung injury by reversing the increases in CD68 (cluster of differentiation 68), MYD88 (myeloid differentiation primary response 88), and TLR4 expression through inhibition of the NF-κB pathway [40]. Molecular docking and molecular dynamics simulations suggested the potential binding of sinensetin to the NF-κB P65 subunit [47]. In LPS-treated mouse tissues, sinensetin reduced phosphorylation of IκBα (inhibitor of NF-κB) at S32/S36 residues, thereby enhancing the binding of IκBα to NF-κB P65 and downregulating NF-κB target gene expression, ultimately ameliorating LPS-induced acute pulmonary inflammation [40].

In human chondrocytes, while higher concentrations of sinensetin (≥5 μg/mL; ~13.4 μM) reduced cell viability, treatment with 1 μg/mL (~2.7 μM) exerted a protective effect against the IL1β-induced cytotoxicity [41]. Sinensetin decreased the secretion of inflammatory mediators such as COX2, IL6, iNOS, MMP13 (matrix metalloproteinase 13), and TNFα in IL1β-treated chondrocytes [41]. It also reversed the IL1β-mediated downregulation of collagen type II [41]. Transcriptome analysis and knockdown experiments identified SERPINA3 (serpin family A member 3) as a factor involved in the IL1β-mediated chondrotoxicity [41]. Sinensetin treatment upregulated SERPINA3 protein (alpha-1-antichymotrypsin) levels in IL1β-treated chondrocytes [41]. IL1β stimulation increased phosphorylation of IκBα and NF-κB P65, leading to nuclear localization of P65, whereas sinensetin suppressed phosphorylation of both proteins [41]. Consistently, sinensetin prevented cartilage degeneration in a rat OA model and reversed changes in SERPINA3, collagen II, and MMP13 expression [41]. Although the sinensetin-mediated upregulation of SERPINA3 is suggested as a mechanism for tis anti-inflammatory effects, no direct evidence has demonstrated that SERPINA3 inhibits NF-κB P65 nuclear translocation. Moreover, the mechanism by which sinensetin upregulates SERPINA3 remains unknown.

### 3.3. MAPK Pathway

In a cultured neurotoxicity model for oxygen–glucose deprivation/reperfusion (OGD/R) injury, sinensetin demonstrated anti-inflammatory and antioxidative effects though MAPK pathways. In human cerebral microvascular endothelial cells (HCMECs) subjected to OGD/R, sinensetin suppressed the OGD/R-induced upregulation of p-P38 (Y182), p-ERK1/2 (T185/T202), and p-JNK (JUN N-terminal kinase) (Y185) levels [42].

Interestingly, sinensetin reduced the IAV (influenza A virus)-induced upregulation of p-P38 in HEK293 cells without affecting the basal level of p-P38 in control HEK293 cells [43]. Recently, it has been reported that MKK6 is a direct target for sinensetin, modulating the MKK6/P38 pathway in non-small cell lung cancer (NSCLC) cells (will discuss later). Furthermore, sinensetin, along with nobiletin, was found to induce the transcriptional activation of CRE (cAMP response element) in the rat pheochromocytoma cell line PC12D [48]. Activation of the cAMP/PKA (protein kinase A)/ERK/CREB signaling by sinensetin has also been observed in cultured hippocampal neurons. Among the pharmacological components of *Citrus reticulata*, sinensetin was identified as the most potent activator of CRE-mediated transcription in rat hippocampal neurons in vitro [49]. Although the levels of p-CREB, p-ERK1/2, and p-PKA substrates were elevated by *C. reticulata* extracts, the specific effects of sinensetin on the individual signaling components have not yet been investigated.

## 4. Anticancer Mechanisms of Action

In addition to anti-inflammatory effects, it has been reported that sinensetin exerts anticancer effects through multiple pathways (Table 3).

### 4.1. β-Catenin Pathway

Anticancer effects of sinensetin through inhibition of the β-catenin pathway have been reported in breast cancer cells. Sinensetin reduced the viability of breast cancer cells (MCF7 and MDA-MB-231) by inducting apoptosis but did not affect the viability of normal mammary epithelial cells [50]. Sinensetin also suppressed invasion and epithelial–mesenchymal transition (EMT) of breast cancer cells, accompanied by the downregulation of β-catenin, LEF1 (lymphatic enhancing factor 1), TCF1 (T-cell factor 1)/TCF7 (transcription factor 7), and TCF3 (transcription factor 3)/TCF7L1 (transcription factor 7 like 1) at both the protein and mRNA levels [50]. Notably, treatment with a WNT (Wingless and Int-1) agonist reversed the sinensetin-mediated inhibition of cell survival, migration, EMT, and the expressions of β-catenin, LEF1, and TCF1/TCF7 proteins [50], suggesting that sinensetin may target WNT signaling or its downstream factors either directly or indirectly.

Regulation of the β-catenin pathway by sinensetin has also been reported in NSCLC cells and xenograft mouse models. Sinensetin inhibited the proliferation and migration of NSCLC cells in vitro and reduced the tumor size in xenograft mice [51]. In NSCLC cells, sinensetin suppressed EMT by downregulating p-AKT (T308), p-GSK3β (S9), and β-catenin protein levels [51]. Sinensetin also enhanced the cytotoxicity of CD8^+^ T-cells and increased the production of pro-inflammatory factors, such as IFNγ, IL2, and TNFα, thereby preventing immune evasion of NSCLC cells [51]. Importantly, treatment with the AKT activator SC79 [57] abrogated the sinensetin-mediated downregulation of p-AKT (T308), p-GSK3β (S9), and β-catenin both in vitro and in vivo, as well as the anti-tumor effects observed in vivo [51], indicating that AKT or its upstream regulators may be direct targets of sinensetin.

### 4.2. PI3K/AKT Pathway

The anticancer effects of sinensetin have also been reported in gallbladder cancer adenocarcinoma (GBAC). In the TJ-GBC2 GBAC cell line, sinensetin induced apoptosis through inhibition of the PI3K/AKT pathway [52]. Sinensetin reduced cell viability and migration in a dose-dependent manner and promoted apoptosis via downregulation of anti-apoptotic BCL2 (B-cell leukemia/lymphoma 2 protein) and upregulation of pro-apoptotic BAX (BCL2-associated X) expressions [52]. In addition, sinensetin inhibited cell migration and invasion by suppressing MMP2 (matrix metalloprotease 2) expression and enhancing MMP9 (matrix metalloprotease 9) expression [52]. Interestingly, sinensetin downregulated the levels of p-PI3K, p-AKT, and PTEN in GBAC cells [52].

Interestingly, potential binding of sinensetin to PI3K has been proposed based on molecular docking studies [58]. Furthermore, intragastric administration of sinensetin reduced the progression of pulmonary fibrosis (PF) in a bleomycin-induced PF mouse model [58]. Although sinensetin administration attenuated the bleomycin-induced upregulation of p-PI3K and p-AKT levels in pulmonary tissues [58], direct evidence of PI3K inhibition by sinensetin has not yet been demonstrated.

### 4.3. VEGF Pathway

The anti-angiogenic effects of sinensetin have been well-documented in a xenograft model with HepG2/C3A tumors. Sinensetin reduced the tumor volume and decreased the expression of neovascularization marker CD31, along with lower levels of VEGF (vascular endothelial growth factor) in tumor tissues [53]. Additionally, sinensetin inhibited both basal- and VEGF-induced proliferation, migration, and tube formation of human umbilical vein endothelial cells (HUVECs) in a dose-dependent manner [53]. Notably, sinensetin also diminished hypoxia-induced *VEGF* mRNA levels in HepG2/C3A, potentially through the downregulation of HIF1α (hypoxia-induced factor 1 α) [53]. Furthermore, sinensetin inhibited the VEGF-induced phosphorylation of VEGFR2 (VEGF receptor 2) and AKT in HUVECs [53]. Molecular docking analysis suggested that sinensetin might bind to the kinase active site of VEGFR2, thereby inhibiting the VEGF/VEGFR2/AKT signaling pathway [53]. However, further in vitro studies are necessary to confirm the binding and inhibition of VEGFR2 by sinensetin. Additional research is also needed to elucidate the mechanism underlying sinensetin-mediated downregulation of HIF1α in cancer cells.

Sinensetin’s effect on the VEGF/VEGFR2/AKT pathway has also been observed in lung adenocarcinoma (LUAD) cells. In these cells, sinensetin induced the expression of the microRNA (miR) *miR-374c-5p*, leading to the negative regulation of the VEGF/VEGFR2/AKT pathway [54]. The *miR-374c-5p* directly binds to the 3’-UTR (untranslated region) of VEGFA, resulting in decreased VEGFA expression [54]. Overexpression of VEGFA or inhibition of *miR-374c-5p* attenuated the anti-tumor effects mediated by sinensetin [54]. Moreover, sinensetin reduced the expression of cancer stem cell markers, including CD44 and CD133 [59], in LUAD cells [54]. In the context of NSCLC, the β-catenin/AKT pathway is implicated in immune evasion, and markers like CD44 and CD133 may play a role in this process [59]. However, the mechanism driving sinensetin-induced expression of *miR-374c-5p* remains to be elucidated and warrants further investigation.

### 4.4. Other Pathways

#### 4.4.1. NRF2 Pathway

A virtual screening identified sinensetin as a potential NRF2 agonist [55]. In carbon tetrachloride (CCl_4_)-exposed HepG2 cells, sinensetin restored the expression of NRF2 and SOD1/2 and malondialdehyde (MDA) contents [55]. Furthermore, sinensetin increased the nuclear expression of NRF2 in CCl_4_-treated rats, which had reduced nuclear NRF2 levels compared to untreated control [55]. Although transient activation of NRF2, in normal cells in response to genotoxic stresses, has chemopreventive roles against carcinogenesis and tumor progression, persistent NRF2 activation may result in cancer progression, resistance to therapies, and poor prognosis [60]. Based on these backgrounds, further investigations are needed to determine the potential effects of sinensetin on the NRF2 pathway in the context of cancer.

#### 4.4.2. P53 Pathway

Sinensetin has been shown to destabilize wild-type P53 in the hepatocellular carcinoma (HCC) cell line HepG2. Sinensetin induced autophagy in HepG2 cells while promoting apoptosis in Hep3B cells in a wild-type P53-dependent manner. In particular, sinensetin induced proteasome-dependent degradation of wild-type P53 in HepG2 cells [56]. It also reduced p-mTOR levels and increased p-AMPK and P27 levels [56]. Moreover, sinensetin promoted the nuclear translocation of P53 in HepG2 cells [56]. Blocking P53 translocation inhibited sinensetin-induced autophagy [56]. Interestingly, molecular docking simulations suggested that sinensetin could bind to the core domain of P53 [56]. The mechanism of sinensetin-induced autophagy is suggested to involve the P53/AMPK/mTOR pathway [56]. Contrary to its effects in HepG2 cells, sinensetin induced apoptosis in P53-null Hep3B cells [56]. Notably, sinensetin demonstrated minimal or no cytotoxic effects on normal human liver epithelial cells [56].

#### 4.4.3. MKK6/P38 Pathway

A recent report [19] by our group suggested MKK6 as a putative target for sinensetin, using four independent techniques based on different principles: (1) in vitro kinase assay: Sinensetin selectively inhibited the phosphorylation of P38α by MKK6 but not MKK3. Purified proteins expressed in *E. coli* were used to exclude contamination from other mammalian kinases or substances. MKK6 kinase activity was measured by detecting p-P38 (T180/Y182) with a specific antibody; (2) in vitro binding assay: The direct binding of sinensetin to purified MKK6 was confirmed by surface plasmon resonance (SPR) analysis, showing an affinity of 66.27 μM in the presence of the substrate (ATP); (3) molecular docking: Although structural analysis of sinensetin-MKK6 complex was not experimentally proven, in silico molecular docking suggested that sinensetin binds to two separate helices (αC and αG) of MKK6. In contrast, sinensetin binds to only one helix (αC) of MKK3; and (4) siRNA-based knockdown experiment: Depletion of MKK6 by siRNA in NSCLC cells abrogated sinensetin-mediated cell growth inhibition. Sinensetin also demonstrated anticancer effects in NSCLC in an MKK6-dependent manner, potentially through induction of P21 expression and G1 phase cell-cycle arrest [19]. Unlike previous studies [61], the AKT or ERK pathway was not commonly regulated by sinensetin in NSCLC cells [19]. Based on molecular docking results, it has been suggested that sinensetin binds to the αG-helix of MKK6 to inhibit its substrate recognition [19]. However, further studies are needed to elucidate the precise molecular mechanisms for the differential binding of sinensetin to MKK6, given that the amino acid sequences of MKK6 and MKK3 have a complete match in their αG-helices and a high degree of similarity in the surrounding sequences [62].

## 5. Immunological Effects of Sinensetin

Tumor-associated macrophages (TAMs) have been implicated in tumor survival, metastasis, and immunity [63,64]. COX2 in TAMs has been reported to promote the survival and metastasis of breast cancer cells, and inhibition of COX2 prevents M2 macrophage polarization [64,65] and suppresses metastasis of breast cancer in a murine cancer model [66]. TAMs comprise M1-like and M2-like TAMs, and targeting M2-like TAMs has been recognized as a potential therapeutic target [67]. Substantial studies suggest potential role(s) of sinensetin in TAMs-mediated tumor progression and immunity (Table 4).

### 5.1. NF-kB Pathway

In macrophages, sinensetin appears to play a role in anti-inflammatory responses. Sinensetin reduced the expression of iNOS and COX2 in the murine macrophage cell line RAW264.7 following LPS treatment [68]. Consistently, LPS-induced mRNA levels for IL1β, IL6, and TNFα were downregulated by sinensetin in RAW264.7 cells [68]. Mechanistically, sinensetin delayed the degradation of IκBα, thereby inhibiting the nuclear translocation of the NF-κB P65 subunit [68].

As mentioned earlier, sinensetin attenuated LPS-induced acute pulmonary inflammation probably through binding to NF-κB P65 subunit [47] and reducing the levels of p-IκBα [40], leading to downregulation of NF-κB target gene expression.

### 5.2. SIRT Pathway

The anti-inflammatory effects of sinensetin have also been reported in an LPS-treated macrophage cell line model. Sinensetin attenuated the LPS-induced lung and liver injuries in mice, as evidenced by a reduction in serum ALT (alanine aminotransferase), IL1β, and TNFα [69]. In the murine macrophage RAW264.7 cells, sinensetin counteracted LPS-mediated modulation of various mRNAs. Specifically, sinensetin reduced LPS-induced upregulation of mRNA expressions for M1-type macrophage markers (*iNos2*, *Cox2*, and *Cd86*), while enhancing the LPS-suppressed mRNA expressions for the M2-type macrophage markers [*Cd206*, *Cd68*, and *Agr1* (arginase 1)] [69]. Molecular docking analysis suggested that sinensetin binds to SIRT1 (sirtuin 1) [69]. Additionally, sinensetin mitigated the LPS-induced downregulation of *Sirt1* and *Nrf2* mRNAs in RAW264.7 cells [69]. Knockdown of *Sirt1* attenuated the sinensetin-mediated NRF2 upregulation [69].

### 5.3. JAK2/STAT Pathway

Sinensetin also attenuated LPS-induced pulmonary inflammation in a mouse model. Along with the NF-κB pathway, sinensetin downregulated the levels of phosphorylated proteins in JAK2 (Janus kinase 2)/STAT pathways, including p-JAK2 (Y1008), p-STAT1 (S727), p-STAT3 (Y705), and p-STAT4 (Y693), in the LPS-treated mice and RAW264.7 macrophage cells [40].

Moreover, sinensetin reduced the expression of *Nos2* mRNA and nitric oxide (NO) production in LPS-treated J774 cells (murine macrophage cells) in a dose-dependent manner [70]. It also reduced LPS-induced levels of COX2 and TNFα proteins and the production of prostaglandin E_2_ (PGE_2_). Interestingly, sinensetin reduced LPS-induced nuclear accumulation of STAT1α (signal transducer and activator of transcription 1α), with little or no effect on LPS-induced translocation of NF-κB P65 in J774 cells [70].

### 5.4. ROS/JNK Pathway

Sinensetin affects the apoptosis and autophagy in human T-cell lymphoma cells. In Jurkat cells, sinensetin induced autophagy by activation of JNK through accumulation of intracellular ROS [61]. The sinensetin-mediated induction of autophagy markers, such as the upregulation of LC3-II and Beclin-1 and downregulation of P62, were observed [61]. In addition, the activation of JNK was abrogated by treatment of N-acetyl cysteine (NAC), an ROS scavenger [61].

### 5.5. Interleukin (IL) Pathways

The immunomodulatory activity of sinensetin was studied in cyclophosphamide (CY)-induced immunosuppressed mice. In CY-treated mice, sinensetin suppressed CY-mediated body weight reduction and organ indices. In addition, sinensetin increased lymphocyte proliferation and secretion of IFNγ, IL2, and IL6 by upregulating the expression of mRNAs for *Ifng*, *Il2*, and *Il6* in CY-treated mice [71].

The TXNIP (thioredoxin-interacting protein)/NLRP2 (NACHT, LRR, and PYD domains-containing protein 2)/Caspase 1/GSDMD (gasdermin D) pathway was also regulated by sinensetin treatment. In both the LPS-treated acute lung injury (ALI) mouse model and cultured RAW264.7 macrophage model, sinensetin attenuated inflammatory responses by inhibiting the dissociation of thioredoxin 1 (TRX1) and TXNIP and reducing the expression of ASC (apoptosis-associated speck-like protein containing a CARD), GSDMD, NLRP3, and pro-caspase 1 [72]. Mechanistically, sinensetin reduced the levels of ROS, and thereby inhibited the dissociation of TRX1 and TXNIP in ALI mice [72]. In addition, molecular docking analysis suggested a potential sinensetin-NLRP3 interaction [72].

## 6. Safety Aspects of Sinensetin

Although intensive investigations on the safety of sinensetin have not been performed yet [7], several studies showed its limited toxicity, especially on normal cells and tissues (Table 5). The maximum non-toxic dose of sinensetin is approximately 1.07 mM in human lung adenocarcinoma cell line A549 after 48 h treatment and assessed by cell counting kit-8 (CCK8) and lactate dehydrogenase (LDH) release assay [43]. However, a study by our group resulted in contradictory results in the antiproliferative effects on A549 cells when they were treated with 60 μM for 72 h in the 3-(4,5-dimethylthiazol-2-yl)-2-5-diphenyltetrazolium bromide (MTT) assay [19]. The maximum non-cytotoxic doses vary from 200 μM on normal human bronchial epithelial cells [51] and mouse primary T-cells [61] and approximately 2.69 μM on human chondrocytes [41]. Notably, a single-dose acute toxicity study in mice with 2000 mg/kg of sinensetin showed no significant toxicity and mortality for 2 weeks [55]. However, more intensive in vivo evaluations, such as toxicity, pharmacokinetic, pharmacodynamic, and biodistribution studies, are required before further clinical development.

## 7. Potential Targets for Sinensetin

For bioactive natural products, the identification of cellular targets (so-called target ID) is crucial for advancing new drug development [73,74,75,76,77]. Target ID is essential not only for understanding the mode of action (MoA) of a given natural product, but also for optimizing its specificity, selectivity, efficacy, and toxicity [73,74,75,76,77]. Although a plethora of technologies for target ID have been developed, the process remains challenging and often requires considerable time to yield validated targets [76]. As a result, many natural compounds still lack identified cellular targets [76].

Although the small molecular weight (372.4 g/mol) of sinensetin aligns well with Lipinski’s rule of five for drug-likeness [78] and it can be efficiently synthesized in high yield [79], its MoA remains elusive due to limited knowledge of its cellular target(s). Several enzymes have been reported as potential targets of sinensetin (Table 6). Molecular docking simulation is widely used to predict the potential interaction between small molecules and cellular target proteins [80]. Molecular docking analyses have suggested various cellular proteins, including AKT [81], AMPKγ [44], caspase 9 [47], COX2 [82], EGFR (epidermal growth factor receptor) [81,82], ERα (estrogen receptor α) [82], fibronetin [83], HIF1α [82], HSP90α (heat shock protein 90α) [81], MAPK3 [82], MYD88 [47], NF-κB P65 [47], NLRP3 [72], P53 [56,81], PI3K [58,81], PPARγ [82], SIRT1 [69], SRC [82], VEGFα [81], and VEGFR2 [53], as potential targets for sinensetin. Although some of these targets were linked to the biological effects of sinensetin, further studies are required to validate the interactions between sinensetin and its targets.

In vitro enzyme assays and spectral analyses, such as fluorescence quenching or circular dichroism (CD) spectra analysis with isolated proteins, are also widely used to screen or analyze small molecule–protein interactions. Using these techniques, several proteins have been identified as potential sinensetin targets, including α-amylase [5], BACE1 [39], BSA (bovine serum albumin) [85], CYP1A2 (cytochrome P450 1A2) [88], α-glucosidase [5,85], IFNγ [90], pancreatic lipase [91], and 15-lipoxygenae [92]. Although some of these were further supported by other in vitro assays or molecular docking simulations [39,85,89,90,91], other studies were based solely on in vitro enzyme assays [5,88,92]. One limitation of in vitro enzyme assays is their proneness to yield pseudo-positives. An example of this limitation is the artifact-prone fluorophore-based in vitro assay for SIRT1 activity, which led to a prolonged debate on the activation of SIRT1 by resveratrol [96,97]. Additionally, the biological consequences of sinensetin–target binding have not been validated in cellular contexts.

Cell-based assays are another method to identify cellular targets for given small molecules. As shown in Table 6, many proteins, such as CYP1A1/B1 [87], CYP3A4 [89], MATE1 (multidrug and toxin extrusion protein 1) [93], P-glycoprotein [18], SLCO1B1 (solute carrier organic anion transporter family member 1B1)/OATP1B1 [94,95], SLCO1B3/OATP1B3 [95], and SLCO2B1/OATP2B1 [95], have been identified as potential targets for sinensetin. Among them, only CYP3A4 was further studied with CD analysis and molecular docking [89]. One limitation of cell-based assays is their inability to differentiate direct effects from indirect or secondary effects. Although most cell-based assays use cells with overexpressed targets, it is difficult to completely exclude the indirect or secondary effects observed as assay readouts. Furthermore, cell-based assays are also susceptible to pseudo-results. For instance, luciferase-based assays sometimes result in false positive signals due to enzymatic interference with luciferase activity by a high proportion of compounds in libraries [98].

BACH1 (BTB and CNC homolog 1), a transcription regulator protein, is one of the most thoroughly studied potential targets for sinensetin. In a rat model, sinensetin ameliorated periodontitis in vivo [84]. BACH1 targeting by sinensetin was investigated using four techniques: (1) in silico molecular docking; (2) cellular thermal displacement assay; (3) knockdown; and (4) overexpression of BACH1. In silico molecular docking identified BACH1 as a sinensetin target [84]. The cellular thermal displacement assay further supported sinensetin-BACH1 binding, as the thermal stability of BACH1 increased in the presence of sinensetin [84]. Mechanistically, sinensetin binding to BACH1 reduced the levels of BACH1 via ubiquitin-dependent proteolysis and prevented its binding to the *HMOX1* (heme oxygenase 1) promoter in periodontal ligament cells (PDLCs) [84]. However, the expression of HMOX1 protein was partially increased by sinensetin, exerting antioxidative responses in PDLCs [84]. Knockdown of BACH1 partially reduced sinensetin-induced HMOX1 expression [84]. Conversely, overexpression of BACH1 enhanced the effect of sinensetin on GSH contents [84]. Interestingly, BACH1 knockdown completely abolished the effects of sinensetin on SOD activity and MDA content in PDLCs [84]. Further studies are needed to investigate the mechanisms of BACH1 ubiquitination by sinensetin and its biological consequences.

As mentioned earlier, our group suggested MKK6 as a putative target for sinensetin [19]. An SPR analysis with purified MKK6 supported direct binding of sinensetin to MKK6 with an affinity of 66.27 μM in the presence of ATP. In vitro kinase assays further demonstrated the selective inhibition of MKK6 but not MKK3 by sinensetin. Molecular docking suggested that sinensetin binds to αC and αG helixes of MKK6, while it binds to only αC helix of MKK3. However, the molecular mechanisms for this selective inhibition of MKK6 remains to be determined. 

## 8. Conclusions and Future Perspectives

**Figure 2:**
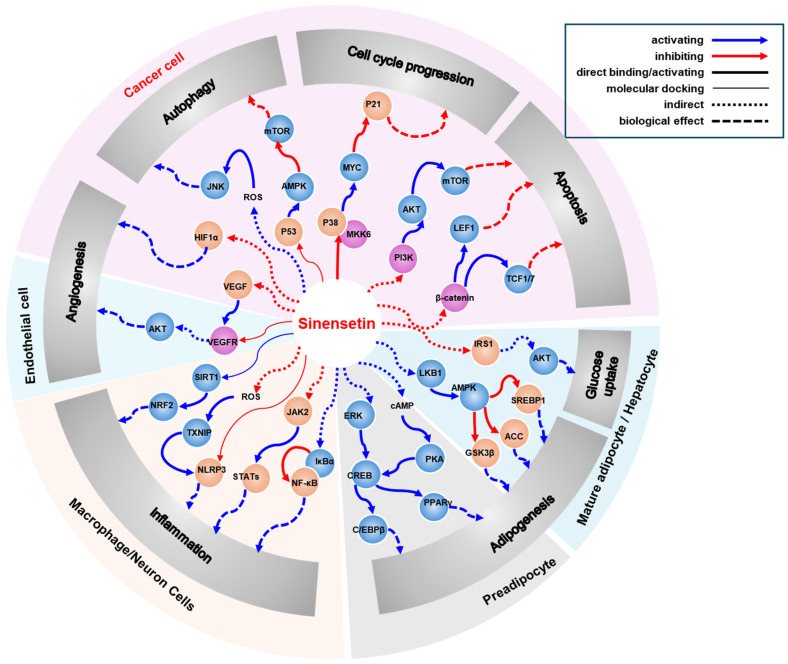
Selected signaling pathways regulated by sinensetin and potential targets of sinensetin.

Sinensetin, a citrus polymethoxylated flavone, has resurfaced as a multi-target natural product with promising anti-inflammatory and anticancer potential by affecting various cellular signaling pathways (Figure 2). In this review, we discussed that sinensetin modulates core inflammatory and oncogenic pathways—including AMPK, NF-κB, MAPK, β-catenin, AKT, P53, and MKK6—to suppress proliferation, migration, and survival signaling while enhancing antioxidant defenses and dampening cytokines such as TNF-α and IL-6. In neuronal and epithelial contexts, sinensetin restrains TLR4/NF-κB activation and supports barrier/autophagy programs via AMPK–ULK1, aligning with protective effects in colitis and OA models. Furthermore, sinensetin attenuated LPS-induced lung inflammation, down-modulated JAK2/STATs phosphorylations, and suppressed ROS-linked TXNIP/NLRP3 inflammasome activation in ALI models. In anticancer aspects, sinensetin inhibited β-catenin/LEF1/TCF signaling, reducing cell viability, EMT, and invasion in breast cancer. Suppression of AKT/GSK3β/β-catenin signaling impaired NSCLC growth. In GBAC cells, it restrained the PTEN/PI3K/AKT pathway, reduced migration, and promoted apoptosis. Beyond tumor cell-intrinsic effects, sinensetin exerts anti-angiogenic actions by lowering VEGF/HIF-1α, inhibiting VEGFR2 and AKT phosphorylations in endothelial cells, and reducing CD31^+^ neovascularization in LUAD xenografts. Immunologically, sinensetin enhanced anti-tumor immunity by boosting CD8^+^ T-cell cytotoxicity and increasing IFNγ/IL-2/TNFα in NSCLC models. Moreover, sinensetin modulated innate/adaptive responses in cyclophosphamide-immunosuppressed mice by improving lymphocyte proliferation and elevating IFNγ/IL-2/IL-6. However, most data are preclinical and often require mid- to high-micromolar concentrations. Therefore, future studies benchmarking other citrus PMFs to delineate structure–activity relationships are required, and this can lead to optimization for potency and selectivity. Closing these gaps will determine whether sinensetin advances from a promising multi-target natural product to a tractable candidate for precision anti-inflammatory, anticancer, and immunotherapy.

Decades-long efforts have been made to identify the cellular targets for sinensetin. As explored in this review, many cellular proteins have been suggested as potential targets for sinensetin. Among these, BACH1 and MKK6 have been intensively studied; however, targeting these proteins alone does not sufficiently explain all the reported biological effects of sinensetin. Additionally, the various biological activities of sinensetin have been observed in mid- and high-micromolar ranges, indicating a need to improve its potency through medicinal chemistry while reducing potential toxic adverse effects. Further validation of potential targets for sinensetin is crucial for advancing our understanding of its MoA and the future development of novel therapeutics.

## Figures and Tables

**Table 1:** Plant source of sinensetin.

Plant Species	Common Name	Major Part(s) Containing Sinensetin	Ref.
*Orthosiphon aristatus* (syn: *O. spicatus*)	Java Tea/ Cat’s Whiskers	Leaves (highest in young leaves)	[7,21]
*Citrus sinensis*	Sweet Orange	Peel	[7,22]
*Citrus reticulata*	Mandarin Tangerine	Peel	[22,23]
*Citrus aurantium*	Bitter Orange	Peel	[22,23]
*Citrus aurantifolia*	Lime	Peel	[22,23]
*Cultivar setoka*	Cheonhyehyang	Peel	[24]
*Citrus limon (Burm.f.)*	Lemon	Peel	[22,23]
*Citrus unshiu Marcov.*	Satsuma mandarin	Peel	[23]
*Citrus medica*	Citron	Peel	[22]
*Citrus maxima*	Shaddock	Peel	[22]
Citrus × paradisi	Grapefruit	Peel	[22]

**Table 2:** Anti-inflammatory signaling pathways regulated by sinensetin.

Pathway/ Target	Model	Dose	Effects	Ref.
AMPK	Primary chondrocytes; OA mouse model (DMM); Colitis rats, Caco-2 cells	10–40 μM [36] 20–80 mg/kg [37]	Protects chondrocytes from apoptosis and matrix loss; restores epithelial barrier and reduces colitis-associated inflammation	[36,37]
NF-κB	SH-SY5Y neuronal cells	10–40 μM [38] 10–100 μM (in vitro) [39]	Reduces oxidative stress, inflammatory mediators, and apoptosis in Aβ-injured neurons (neuroprotective anti-inflammatory effect)	[38,39]
	Acute pulmonary inflammation in mice	25 and 50 mg/kg	Ameliorates lung injury and acute pulmonary inflammation	[40]
	Human chondrocytes; osteoarthritis (OA) rat model	0.1–1 μg/mL (~0.27–2.69 μM) 20 mg/kg	Protects cartilage and reduces IL-1β-driven joint inflammation	[41]
MAPKs	OGD/R-injured human cerebral microvascular endothelial cells (HCMECs)	50 μM	Attenuates neurovascular inflammatory and oxidative responses after ischemia-like insult	[42]
	Influenza A virus (IAV)-infected HEK293 and A549 cells	30–120 μg/mL (80.1–322.3 μM)	Dampens IAV-triggered MAPK activation and associated inflammatory signaling	[43]

**Table 3:** Anticancer signaling pathways regulated by sinensetin.

Pathway/ Target	Model	Dose (μM)	Effects	Ref.
β-catenin	Breast cancer cells (MCF7, MDA-MB-231); NSCLC cells (in vitro and in vivo)	30–120 μM [50] 6.25–200 μM [51] 40 mg/kg [51]	Induces apoptosis and reduces viability of breast cancer cells without affecting normal mammary cells; inhibits NSCLC cell proliferation, migration, and tumor growth	[50,51]
PI3K/AKT	Gallbladder adenocarcinoma (TJ-GBC2)	0.78–200 μM	Dose-dependent reduction in cell viability and migration; promotes apoptosis; inhibits migration and invasion of drug-resistant GBAC cells	[52]
VEGF	HepG2/C3A liver cancer xenografts; HUVECs	40 mg/kg 3–100 μM	Reduces liver tumor volume and neovascularization; blocks endothelial proliferation, migration, and tube formation → strong anti-angiogenic, anti-tumor effect	[53]
	Lung adenocarcinoma (LUAD) cells (in vitro and in vivo)	20–140 μM 40 mg/kg	Inhibits angiogenesis and growth of LUAD tumors; reduces stem-like phenotype linked to chemoresistance and immune evasion	[54]
NRF2	CCl_4_-treated HepG2 cells and rats	12.5–50 μg/mL (33.57–134.28 μM)	Enhances cellular defense against genotoxic/oxidative injury	[55]
P53	HCC cell lines HepG2 (WT p53) and Hep3B (p53-null); normal liver epithelial cells	50 and 100 μM	Autophagic cell death in HepG2 and apoptotic death in Hep3B with minimal cytotoxicity to normal liver epithelial cells	[56]
MKK6/P38	NSCLC cells, xenograft	20–100 μM	Suppresses proliferation and clonogenic survival of NSCLC cells and reduces tumor growth as a selective MKK6 inhibitor	[19]

**Table 4:** Immune signaling regulated by sinensetin.

Pathway/ Target	Model	Dose	Effects	Ref.
NF-κB	RAW264.7 macrophages	25 and 50 μM	Inhibits classical (M1-like) pro-inflammatory activation of macrophages	[68]
SIRT1	LPS-induced lung and liver injury in mice; RAW264.7 macrophages	12.5–50 mg/kg 12.5–50 μM	Less lung/liver damage and systemic cytokines; promotes M2-like anti-inflammatory macrophage phenotype and limits NLRP3 inflammasome formation	[69]
JAK2/STAT	LPS-induced acute lung injury in mice; RAW264.7 and J774 macrophages	25 and 50 mg/kg [40] 25–100 μM [40] 5 and 50 μM [70]	Suppresses JAK2/STAT-dependent inflammatory gene expression and mediator production, alleviating lung inflammation	[40,70]
ROS/JNK	Jurkat human T-cell lymphoma cells	25–200 μM	Induces autophagy-associated cell death in T-cell lymphoma cells	[61]
Interleukins	Cyclophosphamide-induced immunosuppressed mice	20–100 μM 50–200 mg/kg	Increased lymphocyte proliferation and secretion of IL	[71]
	LPS-induced ALI mice; RAW264.7 macrophages	25 and 50 mg/kg 25–100 μM	Inhibits TXNIP/NLRP3/Caspase-1/GSDMD signaling-mediated inflammatory responses improving ALI	[72]

**Table 5:** Studies on the safety of sinensetin.

Safety				Biological Effects			Ref.
Cell line	Maximum Dose	Safety		Cell Line	Effective Doses	Effects	
A549 (human lung adenocarcinoma)	400 μg/mL (1.07 mM)	No significant cytotoxicity up to 48 h in CCK8 and LDH release assays		A549	30–120 μg/mL (80.1–322.3 μM)	Inhibits IAV-induced MAPK signaling and inflammatory responses	[43]
HepG2	200 μg/mL (537.1 μM)	No significant cytotoxicity up to 24 h in MTT assay		HepG2	12.5–50 μg/mL (33.57–134.28 μM)	Protects cells from genotoxic/oxidative injury induced by CCl_4_ treatment	[55]
Mice	2000 mg/kg	No weight loss or internal pathological lesions					
BEAS-2B (normal human bronchial epithelial cells)	200 μM	No significant cytotoxicity up to 12 h in CCK8 assay		A549 and H1299	6.25–200 μM	Reduces cell proliferation and migration	[51]
Mouse primary T-cells	200 μM	Moderate cytotoxicity up to 48 h in CCK8 assay		Jurkat	50 and 100 μM	Induces autophagy-associated cell death	[61]
MCF10A (normal human mammary epithelial cells)	120 μM	No significant cytotoxicity up to 72 h in CCK8 assay		MCF7 and MDA0-MB-231	30–120 μM	Induces apoptosis and reduces viability of breast cancer cells	[50]
NL20 (normal human lung cells)	80 μM	No significant cytotoxicity up to 24 h in MTT assay		A549 AND H1299	20–60 μM	Reduces the cell proliferation and survival	[19]
HCMECs	50 μM	No significant cytotoxicity up to 30 h in CCK8 assay		HCMECs	50 μM	Protects cells from OGD/R-induced inflammation and oxidative stress	[42]
SH-SY5Y (human neuroblastoma)	40 μM	No significant cytotoxicity up to 24 h in CCK8 and LDH release assays		SH-SY5Y	10–40 μM	Protects cells from the Aβ-induced toxicity	[38]
Primary mice chondrocytes	40 μM	No significant cytotoxicity up to 48 h in CCK8 assay		Primary mice chondrocytes	10–40 μM	Inhibits apoptosis in the TBHP-treated cells	[36]
Human chondrocytes	1 μg/mL (~2.69 μM)	No significant cytotoxicity up to 72 h in CCK8 assay		Human chondrocytes	0.1–1 μg/mL (~0.27–2.69 μM)	Protects cells from IL-1β-induced inflammation	[41]

**Table 6:** Potential targets for sinensetin.

Target Name	Evidence	IC_50_ (μM)	Activity	Biological Effects	Ref.
Amylase, α-	In vitro enzyme assay	3.03	Anti-diabetes	Not determined	[5]
AKT1	Molecular docking	NA	Anti-blood-stasis	Not determined	[81]
AMPKγ	Molecular docking	NA	Anti-adipogenesis	Reversed the PA-induced accumulation of lipid, TG content, and glucose uptake in HepG2 cells	[44]
BACH1	Molecular docking	NA	Anti-inflammation	Reduced the periodontitis in a rat model Prevented BACH1 binding to the HMOX1 promoter to induce HMOX1 expression BACH1 knockdown partially reduced the sinensetin-induced HMOX1 expression in PDLCs BACH1 overexpression increased the effect of sinensetin on GSH contents	[84]
	Cellular thermal displacement assay	NA			
	Knockdown in cells	NA			
	Overexpression in cells	NA			
BACE1	In vitro enzyme assay	63	Anti-Alzheimer’s disease	Not determined	[39]
	FRET substrate decomposition assay	NA			
	Molecular docking	NA			
BSA (AGEs formation)	In vitro enzyme assay	342	Anti-diabetes	Not determined	[85]
	Circular dichroism (CD) analysis	NA			
	Molecular docking	NA			
CBS	Molecular docking	NA	Anti-adipogenesis	Reversed the PA-mediated lipid accumulation, TG contents, and glucose uptake in HepG2 cells	[86]
CYP1A1/B1	Cell-based assay	NA	Antiproliferation	Reduced the proliferation of CYP1A1/B1 expressed cells	[87]
CYP1A2	In vitro enzyme assay	40.2	Unknown	Not determined	[88]
CYP3A4	Molecular docking	NA	Hepatoprotection	Decreased the dronedarone-induced cytotoxicity in CYP3A4-overexpressed HepG2 cells	[89]
	CD analysis	NA			
	Cell-based assay	NA			
Caspase 9	Molecular docking	NA	Anti-inflammation	Reduced the LPS-induced acute lung injury in a mouse model [40]	[47]
COX2	Molecular docking	NA	Anti-inflammation	Alleviated the DSS-induced UC in mice	[82]
EGFR	Molecular docking	NA	Anti-blood-stasis	Not determined	[81]
			Anti-inflammation	Alleviated the DSS-induced UC in mice	[82]
ERα	Molecular docking	NA	Anti-inflammation	Alleviated the DSS-induced UC in mice	[82]
Fibronectin	Molecular docking	NA	Potentially anticancer	Not determined	[83]
Glucosidase, α-	In vitro enzyme assay	1.77	Anti-diabetes	Not determined	[5]
	In vitro enzyme assay	746		Not determined	[85]
	Fluorescence spectra analyses	NA			
	Molecular docking	NA			
HIF1α	Molecular docking	NA	Anti-inflammation	Alleviated the DSS-induced UC in mice	[82]
HSP90α	Molecular docking	NA	Anti-blood-stasis	Not determined	[81]
IFNγ	Fluorescence quenching analysis	NA	Anticancer	Synergistically induced anticancer activity in HepG2 cells via enhancing the expression of caspase 3 mRNA and protein	[90]
	CD analysis				
	Molecular docking				
Lipase, pancreatic	In vitro enzyme assay	526	Anti-obesity	Prevented body weight gain in mice by reducing the blood TG and glycerol contents and hepatic lipids	[91]
	Molecular docking	NA			
Lipoxygenase, 15-	In vitro enzyme assay	74	Anti-atherosclerosis?	Not determined	[92]
MAPK3	Molecular docking	NA	Anti-inflammation	Alleviated the DSS-induced UC in mice	[82]
MATE1	Cell-based assay	14.06	nephroprotection	Exacerbated cisplatin-induced renal injury in mice	[93]
MKK6	In vitro enzyme assay	<40	Anticancer	Reduced the proliferation of NSCLC cells with G1 arrest MKK6 knockdown abolished the sinensetin-mediated inhibition of cell proliferation and survival	[19]
	In vitro binding (SPR) assay	K_D_: 66.27			
	Molecular docking	NA			
	Knockdown in cells	NA			
MyD88	Molecular docking	NA	Anti-inflammation	Reduced the LPS-induced acute lung injury in a mouse model [40]	[47]
NF-κB P65	Molecular docking	NA	Anti-inflammation	Reduced the LPS-induced acute lung injury in a mouse model [40]	[47]
NLRP3	Molecular docking	NA	Anti-inflammation	Ameliorated the LPS-induced acute lung injury in mice and reduced the LPS-induced inflammatory responses in macrophages	[72]
P53	Molecular docking	NA	Anticancer	Destabilized P53 to induce autophagy in an HCC cell line expressing wild-type P53, while inducing apoptosis in a P53-mutant HCC cell line	[56]
		NA	Anti-blood-stasis	Not determined	[81]
PI3K	Molecular docking	NA	Anti-fibrosis	Reduced the pulmonary fibrosis progression	[58]
		NA	Anti-blood-stasis	Not determined	[81]
P-glycoprotein	Cell-based assay	18.9	-	Inhibited P-gp-mediated digoxin transport in MDR1-MDCKII cells	[18]
		NA	Chemosensitization	Sensitizes cancer cells to chemotherapeutics by reducing the levels of P-glycoprotein or inhibiting P-glycoprotein activity	[8,15,16,17]
PPARγ	Molecular docking	NA	Anti-inflammation	Alleviated the DSS-induced UC in mice	[82]
SIRT1	Molecular docking	NA	Anti-inflammation	Reduced the LPS-induced inflammation in mice Reduced the LPS-induced M1-type polarization of macrophages	[69]
SLCO1B1/OATP1B1	Cell-based assay	40.15	Hepatoprotection	Reduces the bosentan-induced liver injury through inhibiting SLCO1B1-mediated bosentan uptake in SLCO1B1-overexpressing HEK293 cells	[94]
	Cell-based assay	8.0	Potential risk of food–drug interactions	Reduced the uptake of 8-fluorescein-cAMP in the SLCO1B1-overexpressing HEK293 cells	[95]
SLCO1B3/OATP1B3	Cell-based assay	21.1	Potential risk of food–drug interactions	Reduced the uptake of 8-fluorescein-cAMP in the SLCO1B3-overexpressing HEK293 cells	[95]
SLCO2B1/OATP2B1	Cell-based assay	5.4	Potential risk of food–drug interactions	Reduced the uptake of 4’,5’-dibromofluorescein in the SLCO2B1-overexpressing HEK293 cells	[95]
SRC	Molecular docking	NA	Anti-inflammation	Alleviated the DSS-induced UC in mice	[82]
VEGFα	Molecular docking	NA	Anti-blood-stasis	Not determined	[81]
VEGFR2	Molecular docking	NA	Anti-angiogenesis	Reduced the angiogenesis of endothelial cells	[53]

## Data Availability

No new data were created or analyzed in this study. Data sharing is not applicable to this article.

## Abbreviations

The following abbreviations are used in this manuscript:

ABCG2	ATP-binding cassette subfamily G member 2
Aβ	Amyloid β
ACC	Acetyl-CoA carboxylase
AD	Alzheimer’s disease
ADORA	Adenosine receptor
ALI	Acute lung injury
ALT	Alanine aminotransferase
AMPK	AMP-activated protein kinase
aP2	Adipocyte protein 2
ARE	Antioxidant responsive element
ARG1	Arginase 1
ASC	Apoptosis-associated speck-like protein containing a CARD
BACH1	BTB and CNC homology 1
BACE1	Beta-secretase 1
BAX	BCL2-associated X
BCL2	B-cell leukemia/lymphoma 2 protein
BMAL1	Basic helix-loop-helix ARNT like 1
BSA	Bovine serum albumin
cAMP	Cyclic adenosine monophosphate
CAT	Catalase
CBS	Cystathionine-β-synthase
CCK8	Cell counting kit-8
CD	Circular dichroism
CD68	Cluster of differentiation 68
C/EBPα	CCAAT/enhancer-binding protein α
cGMP	Cyclic guanosine monophosphate
CLOCK1	Circadian locomotor output cycles protein keput
COX2	Cyclooxygenase-2
CRE	cAMP response element
CREB	cAMP-response element-binding protein
CXCL10	C-X-C motif chemokine 10
CY	Cyclophosphamide
CYP	Cytochrome P450
DMM	Destabilization of medial meniscus
ECM	Extracellular matrix
EGFP	Enhanced green fluorescent protein
EMT	Epithelial–mesenchymal transition
EGFR	Epidermal growth factor receptor
ERα	Estrogen receptor α
ERK	Extracellular signal-regulated kinase
GBAC	Gallbladder cancer adenocarcinoma
GPX1	Glutathione peroxidase-1
GSDMD	Gasdermin D
GSK3β	Glycogen synthase kinase 3β
GSH	Glutathione
HCC	Hepatocellular carcinoma
HCMECs	Human cerebral microvascular endothelial cells
HIF1α	Hypoxia-induced factor 1 α
HMGCR	HMG-CoA reductase
HMOX1	Heme oxygenase 1
HSP90α	Heat shock protein 90α
HUVEC	Human umbilical vein endothelial cell
IAV	Influenza A virus
IBMX	3-isobutyl-1-methylxanthine
IFNγ	Interferon γ
IL	Interleukin
IκBα	Inhibitor of NF-κB
iNOS	Inducible NO synthase
IRS	Insulin receptor substrate
JAK2	Janus kinase 2
JNK	JUN N-terminal kinase
kdrl	Kinase insert domain receptor-like
LC3	Microtubule-associated protein 1A/1B light chain 3
LEF1	Lymphatic enhancing factor 1
LKB1	Liver kinase B1
LPS	Lipopolysaccharide
LDH	Lactate dehydrogenase
LUAD	Lung adenocarcinoma
miR	MicroRNA
MAPK	Mitogen-activated protein kinase
MCP1	Monocyte chemoattractant protein-1
MDA	Malondialdehyde
MKK6	Mitogen-activated protein kinase kinase 6
MoA	Mode of action
MMP	Matrix metalloproteinase
mTOR	Mammalian target of rapamycin
MTT	3-(4,5-dimethylthiazol-2-yl)-2,5-diphenyltetrazolium bromide
MyD88	Myeloid differentiation primary response 88
MYOD	Myoblast determination
NAC	N-acetyl cysteine
NF-κB	Nuclear factor κB-light-chain-enhancer of activated B cells
NLRP	NACHT, LRR, and PYD domains-containing protein
NO	Nitric oxide;
NQO1	NAD(P)H:quinone oxidoreductase 1
NRF2	Nuclear factor erythroid 2-related factor 2
NSCLC	Non-small cell lung cancer
OA	Osteoarthritis
OGD/R	Oxygen–glucose deprivation/reperfusion
PA	Palmitate
PARP	Poly(ADP-ribose) polymerase
PDLCs	Periodontal ligament cells
PE	Phenylephrine
PF	Pulmonary fibrosis
PI3K	Phosphoinositide 3-kinase
PKA	Protein kinase A
PMFs	Polymethoxylated flavones
PPARγ	Peroxisome proliferator-activated receptor γ
PTEN	Phosphatase and tensin homolog
ROR	Receptor tyrosine kinase-like orphan receptor
ROS	Reactive oxygen species
SERPINA3	Serpin family A member 3
sGC	Soluble guanylate cyclase
SIRT1	Sirtuin 1
SLCO1B1	Solute carrier organic anion transporter family member
SOD	Superoxide dismutase
SPR	Surface plasmon resonance
SREBP1c	Sterol regulatory element-binding protein 1c
SREBP2	Sterol regulatory element-binding protein 2
STAT	Signal transducer and activator of transcription
Target ID	Target identification
TBHP	Tert-butyl hydroperoxide
TCF1	T-cell factor 1
TCF3	Transcription factor 3
TCF7	Transcription factor 3
TCF7L1	Transcription factor 7 like 1
TG	Triglyceride
TLR4	Toll-like receptor 4
TNBS	2,4,6-trinitrobenzene sulfonic acid
TNFα	Tumor necrosis factor-α
TRX1	Thioredoxin 1
TXNIP	Thioredoxin-interacting protein
ULK1	Unc-51-like kinase 1
UTR	Untranslated region
VEGF	Vascular endothelial growth factor
VEGFR	VEGF receptor;
WNT	Wingless and Int-1

## References

[1] Atanasov A.G., Zotchev S.B., Dirsch V.M., Orhan I.E., Banach M., Rollinger J.M., Barreca D., Weckwerth W., Bauer R., Bayer E.A. (2021). Natural Products in Drug Discovery: Advances and Opportunities. Nat. Rev. Drug Discov..

[2] Awale S., Tezuka Y., Banskota A.H., Kadota S. (2003). Siphonols A–E: Novel Nitric Oxide Inhibitors from *Orthosiphon stamineus* of Indonesia. Bioorg. Med. Chem. Lett..

[3] Beaux D., Fleurentin J., Mortier F. (1999). Effect of Extracts of *Orthosiphon stamineus* Benth, *Hieracium pilosella* L., *Sambucus nigra* L. and *Arctostaphylos uva-ursi* (L.) Spreng. in Rats. Phytother. Res..

[4] Adam Y., Somchit M.N., Sulaiman M.R., Nasaruddin A.A., Zuraini A., Bustamam A.A., Zakaria Z.A. (2009). Diuretic Properties of *Orthosiphon stamineus* Benth. J. Ethnopharmacol..

[5] Mohamed E.A.H., Siddiqui M.J.A., Ang L.F., Sadikun A., Chan S.H., Tan S.C., Asmawi M.Z., Yam M.F. (2012). Potent α-Glucosidase and α-Amylase Inhibitory Activities of Standardized 50% Ethanolic Extracts and Sinensetin from *Orthosiphon stamineus* Benth as Anti-Diabetic Mechanism. BMC Complement. Altern. Med..

[6] Mohamed E.A.H., Yam M.F., Ang L.F., Mohamed A.J., Asmawi M.Z. (2013). Antidiabetic Properties and Mechanism of Action of *Orthosiphon stamineus* Benth Bioactive Sub-Fraction in Streptozotocin-Induced Diabetic Rats. J. Acupunct. Meridian Stud..

[7] Jie L.H., Jantan I., Yusoff S.D., Jalil J., Husain K. (2021). Sinensetin: An Insight on Its Pharmacological Activities, Mechanisms of Action and Toxicity. Front. Pharmacol..

[8] Mertens-Talcott S.U., Castro W.V.D., Manthey J.A., Derendorf H., Butterweck V. (2007). Polymethoxylated Flavones and Other Phenolic Derivates from Citrus in Their Inhibitory Effects on P-Glycoprotein-Mediated Transport of Talinolol in Caco-2 Cells. J. Agric. Food Chem..

[9] Sergeev I.N., Li S., Colby J., Ho C.-T., Dushenkov S. (2006). Polymethoxylated Flavones Induce Ca^2+^-Mediated Apoptosis in Breast Cancer Cells. Life Sci..

[10] Tezuka Y., Stampoulis P., Banskota A.H., Awale S., Tran K.Q., Saiki I., Kadota S. (2008). Constituents of the Vietnamese Medicinal Plant *Orthosiphon stamineus*. Chem. Pharm. Bull..

[11] Gan R., Liu Y., Li H., Xia Y., Guo H., Geng F., Zhuang Q., Li H., Wu D. (2024). Natural Sources, Refined Extraction, Biosynthesis, Metabolism, and Bioactivities of Dietary Polymethoxyflavones (PMFs). Food Sci. Hum. Wellness.

[12] Lam I.K., Alex D., Wang Y., Liu P., Liu A., Du G., Lee S.M.Y. (2012). In Vitro and in Vivo Structure and Activity Relationship Analysis of Polymethoxylated Flavonoids: Identifying Sinensetin as a Novel Antiangiogenesis Agent. Mol. Nutr. Food Res..

[13] Al-Majmaie S., Nahar L., Rahman M.M., Sharples G.P., Sarker S.D. (2025). Antimicrobial Potential of the Leaves of *Citrus grandis* (L.) Osbeck Collected from Iraq: Bioassay-Guided Isolation of Sinensetin as the Anti-MRSA Compound. Fitoterapia.

[14] Zhao P., Zhang C., Che Y., Zhang L., Lin H., Su Z., Kang Q., Zhang Z., Peng X., Wang T. (2025). Identification of Anti-SARS-CoV-2 Compounds from Qingwen Zhike Prescription and Exploration of Their Underlying Mechanism by UPLC-Q-Exactive Orbitrap MS, High-Throughput Screening Assays and Transmission Electron Microscopy. J. Pharm. Biomed. Anal..

[15] Choi C.-H., Sun K.-H., An C.-S., Yoo J.-C., Hahm K.-S., Lee I.-H., Sohng J.-K., Kim Y.-C. (2002). Reversal of P-Glycoprotein-Mediated Multidrug Resistance by 5,6,7,3′,4′-Pentamethoxyflavone (Sinensetin). Biochem. Biophys. Res. Commun..

[16] Choi C.-H., Kim J.-H., Kim S.-H. (2004). Reversal of P-Glycoprotein-Mediated MDR by 5,7,3′,4′,5′-Pentamethoxyflavone and SAR. Biochem. Biophys. Res. Commun..

[17] Ohtani H., Ikegawa T., Honda Y., Kohyama N., Morimoto S., Shoyama Y., Juichi M., Naito M., Tsuruo T., Sawada Y. (2007). Effects of Various Methoxyflavones on Vincristine Uptake and Multidrug Resistance to Vincristine in P-Gp-Overexpressing K562/ADM Cells. Pharm. Res..

[18] Bai J., Zhao S., Fan X., Chen Y., Zou X., Hu M., Wang B., Jin J., Wang X., Hu J. (2019). Inhibitory Effects of Flavonoids on P-Glycoprotein in Vitro and in Vivo: Food/Herb-Drug Interactions and Structure–Activity Relationships. Toxicol. Appl. Pharmacol..

[19] Xie X., Shin Y.R., Kim T.-S., Seong Y.-S., Yi Y.W., JoonKim D. (2025). Identification of Sinensetin as a Selective Inhibitor for Mitogen-Activated Protein Kinase Kinase 6 and an Anticancer Agent for Non-Small Cell Lung Cancer. Am. J. Cancer Res..

[20] Li Y., Li W., Ye Z., Ji C., Zhou Z. (2024). Antioxidant, Anti-Inflammatory, and Anticancer Activities of Five Citrus Peel Essential Oils. Antioxidants.

[21] Mujahid R., Wahyono S., Jokopriyambodo W., Budiarti M., Widodo H. (2024). Sinensetin Content in Java Tea (*Orthosiphon aristatus* (Blume) Miq.) Based on Age Level of Leaf. AIP Conf. Proc..

[22] Green C.O., Wheatley A.O., Osagie A.U., Morrison E.Y.S.A., Asemota H.N. (2007). Determination of Polymethoxylated Flavones in Peels of Selected Jamaican and Mexican Citrus (*Citrus* spp.) Cultivars by High-performance Liquid Chromatography. Biomed. Chromatogr..

[23] Wang X., Zhan J., Ho C.-T., Li S. (2022). Synthetic Pathways of Sinensetin and Derivatives as an Alternate Source in Biological Activity Study. J. Food Bioact..

[24] Nakanishi M., Hino M., Yoshimura M., Amakura Y., Nomoto H. (2019). Identification of Sinensetin and Nobiletin as Major Antitrypanosomal Factors in a Citrus Cultivar. Exp. Parasitol..

[25] Huang Q., Liu J., Peng C., Han X., Tan Z. (2024). Hesperidin Ameliorates H_2_O_2_-Induced Bovine Mammary Epithelial Cell Oxidative Stress via the Nrf2 Signaling Pathway. J. Anim. Sci. Biotechnol..

[26] Lou J., Wu F., He W., Hu R., Cai Z., Chen G., Zhao W., Zhang Z., Si Y. (2024). Hesperidin Activates Nrf2 to Protect Cochlear Hair Cells from Cisplatin-Induced Damage. Redox Rep. Commun. Free Radic. Res..

[27] Gao J., Yuan L., Jiang H., Li G., Zhang Y., Zhou R., Xian W., Zou Y., Du Q., Zhou X. (2024). Naringenin Modulates Oxidative Stress and Lipid Metabolism: Insights from Network Pharmacology, Mendelian Randomization, and Molecular Docking. Front. Pharmacol..

[28] Flores-Peña R., Monroy-Ramirez H.C., Caloca-Camarena F., Arceo-Orozco S., Salto-Sevilla J.A., Galicia-Moreno M., Armendariz-Borunda J. (2025). Naringin and Naringenin in Liver Health: A Review of Molecular and Epigenetic Mechanisms and Emerging Therapeutic Strategies. Antioxidants.

[29] Li J., Xu M., Wu N., Wu F., Chen J., Xu X., Tan F., Liu Y. (2025). Effects of Citrus-Derived Diosmetin on Melanoma: Induction of Apoptosis and Autophagy Mediated by PI3K/Akt/mTOR Pathway Inhibition. Anti-Cancer Agents Med. Chem..

[30] Jeong S.H., Kim H.H., Ha S.E., Park M.Y., Bhosale P.B., Abusaliya A., Park K.I., Heo J.D., Kim H.W., Kim G.S. (2022). Flavones: Six Selected Flavones and Their Related Signaling Pathways That Induce Apoptosis in Cancer. Int. J. Mol. Sci..

[31] Zhao C., Lai W., Li Y., Hong K., Xu Y. (2025). Potential and Mechanism of Nobiletin in Diabetes Mellitus and Associated Complications. Pharmaceuticals.

[32] Kim E., Mawatari K., Yoo S.-H., Chen Z. (2023). The Circadian Nobiletin-ROR Axis Suppresses Adipogenic Differentiation and IκBα/NF-κB Signaling in Adipocytes. Nutrients.

[33] Arafa E.-S.A., Shurrab N.T., Buabeid M.A. (2021). Therapeutic Implications of a Polymethoxylated Flavone, Tangeretin, in the Management of Cancer via Modulation of Different Molecular Pathways. Adv. Pharmacol. Pharm. Sci..

[34] Li Y.R., Li S., Ho C.-T., Chang Y.-H., Tan K.-T., Chung T.-W., Wang B.-Y., Chen Y.-K., Lin C.-C. (2015). Tangeretin Derivative, 5-Acetyloxy-6,7,8,4′-Tetramethoxyflavone Induces G2/M Arrest, Apoptosis and Autophagy in Human Non-Small Cell Lung Cancer Cells In Vitro and In Vivo. Cancer Biol. Ther..

[35] Navajas-Porras B., Bosch-Sierra N., Valle C.G., Salazar J.D., Marqués-Cardete R., Sáez G., Morillas C., Bañuls C. (2025). Effects of a Flavonoid-Enriched Orange Juice on Antioxidant Capacity, Lipid Profile, and Inflammation in Obese Patients: A Randomized Placebo-Controlled Trial. Food Res. Int..

[36] Zhou W., Shi Y., Wang H., Yu C., Zhu H., Wu A. (2021). Sinensetin Reduces Osteoarthritis Pathology in the Tert-Butyl Hydroperoxide-Treated Chondrocytes and the Destabilization of the Medial Meniscus Model Mice via the AMPK/mTOR Signaling Pathway. Front. Pharmacol..

[37] Xiong Y., Deng Z., Liu J., Qiu J., Guo L., Feng P., Sui J., Chen D., Guo H. (2019). Enhancement of Epithelial Cell Autophagy Induced by Sinensetin Alleviates Epithelial Barrier Dysfunction in Colitis. Pharmacol. Res..

[38] Zhi Z., Tang X., Wang Y., Chen R., Ji H. (2021). Sinensetin Attenuates Amyloid Beta25-35-Induced Oxidative Stress, Inflammation, and Apoptosis in SH-SY5Y Cells Through the TLR4/NF-κB Signaling Pathway. Neurochem. Res..

[39] Youn K., Yu Y., Lee J., Jeong W.-S., Ho C.-T., Jun M. (2017). Polymethoxyflavones: Novel β-Secretase (BACE1) Inhibitors from Citrus Peels. Nutrients.

[40] Xu Z., Wang K., Hu H., Hu Y., Huang J., Luo Z. (2025). Sinensetin Attenuates LPS-Induced Acute Pulmonary Inflammation in Mice and RAW264.7 Cells by Modulating NF-κB P65-Mediated Immune Resistance and STAT3-Mediated Tissue Resilience. Int. Immunopharmacol..

[41] Liu Z., Liu R., Wang R., Dai J., Chen H., Wang J., Li X. (2022). Sinensetin Attenuates IL-1β-Induced Cartilage Damage and Ameliorates Osteoarthritis by Regulating SERPINA3. Food Funct..

[42] Yang D., Yang R., Shen J., Huang L., Men S., Wang T. (2022). Sinensetin Attenuates Oxygen–Glucose Deprivation/Reperfusion-induced Neurotoxicity by MAPK Pathway in Human Cerebral Microvascular Endothelial Cells. J. Appl. Toxicol..

[43] Li J., Jie X., Liang X., Chen Z., Xie P., Pan X., Zhou B., Li J. (2020). Sinensetin Suppresses Influenza a Virus-Triggered Inflammation through Inhibition of NF-κB and MAPKs Signalings. BMC Complement. Med. Ther..

[44] Kang S., Shin H., Ko H., Kim S. (2012). Effects of Sinensetin on Lipid Metabolism in Mature 3T3-L1 Adipocytes. Phytother. Res..

[45] Zhang Y., Chen H., Li R., Sterling K., Song W. (2023). Amyloid β-Based Therapy for Alzheimer’s Disease: Challenges, Successes and Future. Signal Transduct. Target. Ther..

[46] Chen C.-H., Zhou W., Liu S., Deng Y., Cai F., Tone M., Tone Y., Tong Y., Song W. (2012). Increased NF-κB Signalling up-Regulates BACE1 Expression and Its Therapeutic Potential in Alzheimer’s Disease. Int. J. Neuropsychopharmacol..

[47] Huang J., Xu Z., Li J., He X., Huang X., Shen X., Luo Z. (2024). Systems Pharmacology-Based Dissection of Potential Mechanisms of Exocarpium Citri Grandis for the Treatment of Chronic Bronchitis. Arab. J. Chem..

[48] Nagase H., Omae N., Omori A., Nakagawasai O., Tadano T., Yokosuka A., Sashida Y., Mimaki Y., Yamakuni T., Ohizumi Y. (2005). Nobiletin and Its Related Flavonoids with CRE-Dependent Transcription-Stimulating and Neuritegenic Activities. Biochem. Biophys. Res. Commun..

[49] Kawahata I., Yoshida M., Sun W., Nakajima A., Lai Y., Osaka N., Matsuzaki K., Yokosuka A., Mimaki Y., Naganuma A. (2013). Potent Activity of Nobiletin-Rich Citrus Reticulata Peel Extract to Facilitate cAMP/PKA/ERK/CREB Signaling Associated with Learning and Memory in Cultured Hippocampal Neurons: Identification of the Substances Responsible for the Pharmacological Action. J. Neural Transm..

[50] Zhu S., Meng L., Wei P., Gu G., Duan K. (2024). Sinensetin Suppresses Breast Cancer Cell Progression via Wnt/β-Catenin Pathway Inhibition. Transl. Cancer Res..

[51] Shi Z., Shen Y., Liu X., Zhang S. (2024). Sinensetin Inhibits the Movement Ability and Tumor Immune Microenvironment of Non-small Cell Lung Cancer Through the Inactivation of AKT/Β-Catenin Axis. J. Biochem. Mol. Toxicol..

[52] Huang B., Zhai M., Qin A., Wu J., Jiang X., Qiao Z. (2020). Sinensetin Flavone Exhibits Potent Anticancer Activity against Drug-Resistant Human Gallbladder Adenocarcinoma Cells by Targeting PTEN/PI3K/AKT Signalling Pathway, Induces Cellular Apoptosis and Inhibits Cell Migration and Invasion. J. BU Off. J. Balk. Union Oncol..

[53] Li X., Li Y., Wang Y., Liu F., Liu Y., Liang J., Zhan R., Wu Y., Ren H., Zhang X. (2022). Sinensetin Suppresses Angiogenesis in Liver Cancer by Targeting the VEGF/VEGFR2/AKT Signaling Pathway. Exp. Ther. Med..

[54] Ji T., Ye L., Xi E., Liu Y., Wang X., Wang S. (2024). Sinensetin Inhibits Angiogenesis in Lung Adenocarcinoma via the miR-374c-5p/VEGF-A/VEGFR-2/AKT Axis. Cell Biochem. Biophys..

[55] Liu S., Qin H.-H., Ji X.-R., Gan J.-W., Sun M.-J., Tao J., Tao Z.-Q., Zhao G.-N., Ma B.-X. (2023). Virtual Screening of Nrf2 Dietary-Derived Agonists and Safety by a New Deep-Learning Model and Verified In Vitro and In Vivo. J. Agric. Food Chem..

[56] Kim S.M., Ha S.E., Lee H.J., Rampogu S., Vetrivel P., Kim H.H., Saralamma V.V.G., Lee K.W., Kim G.S. (2020). Sinensetin Induces Autophagic Cell Death through P53-Related AMPK/mTOR Signaling in Hepatocellular Carcinoma HepG2 Cells. Nutrients.

[57] Zhu J., Wu Y., Wu D., Luo W.-F., Zhang Z., Liu C. (2019). SC79, a Novel Akt Activator, Protects Dopaminergic Neuronal Cells from MPP+ and Rotenone. Mol. Cell. Biochem..

[58] Xu Y., Hang W.-L., Zhou X.-M., Wu Q. (2021). Exploring the Mechanism Whereby Sinensetin Delays the Progression of Pulmonary Fibrosis Based on Network Pharmacology and Pulmonary Fibrosis Models. Front. Pharmacol..

[59] Sarabia-Sánchez M.A., Tinajero-Rodríguez J.M., Ortiz-Sánchez E., Alvarado-Ortiz E. (2024). Cancer Stem Cell Markers: Symphonic Masters of Chemoresistance and Immune Evasion. Life Sci..

[60] Pouremamali F., Pouremamali A., Dadashpour M., Soozangar N., Jeddi F. (2022). An Update of Nrf2 Activators and Inhibitors in Cancer Prevention/Promotion. Cell Commun. Signal..

[61] Tan K.-T., Lin M.-X., Lin S.-C., Tung Y.-T., Lin S.-H., Lin C.-C. (2019). Sinensetin Induces Apoptosis and Autophagy in the Treatment of Human T-Cell Lymphoma. Anti-Cancer Drugs.

[62] Juyoux P., Galdadas I., Gobbo D., von Velsen J., Pelosse M., Tully M., Vadas O., Gervasio F.L., Pellegrini E., Bowler M.W. (2023). Architecture of the MKK6-P38α Complex Defines the Basis of MAPK Specificity and Activation. Science.

[63] Li M., He L., Zhu J., Zhang P., Liang S. (2022). Targeting Tumor-Associated Macrophages for Cancer Treatment. Cell Biosci..

[64] Pan Y., Yu Y., Wang X., Zhang T. (2020). Tumor-Associated Macrophages in Tumor Immunity. Front. Immunol..

[65] Gan L., Qiu Z., Huang J., Li Y., Huang H., Xiang T., Wan J., Hui T., Lin Y., Li H. (2016). Cyclooxygenase-2 in Tumor-Associated Macrophages Promotes Metastatic Potential of Breast Cancer Cells through Akt Pathway. Int. J. Biol. Sci..

[66] Na Y.-R., Yoon Y.-N., Son D.-I., Seok S.-H. (2013). Cyclooxygenase-2 Inhibition Blocks M2 Macrophage Differentiation and Suppresses Metastasis in Murine Breast Cancer Model. PLoS ONE.

[67] Wang S., Wang J., Chen Z., Luo J., Guo W., Sun L., Lin L. (2024). Targeting M2-Like Tumor-Associated Macrophages Is a Potential Therapeutic Approach to Overcome Antitumor Drug Resistance. npj Precis. Oncol..

[68] Shin H.-S., Kang S.-I., Yoon S.-A., Ko H.-C., Kim S.-J. (2012). Sinensetin Attenuates LPS-Induced Inflammation by Regulating the Protein Level of IκB-α. Biosci. Biotechnol. Biochem..

[69] Lin L., Deng K., Gong Z., Fan H., Zhang D., Lu G. (2023). Sinensetin Attenuated Macrophagic NLRP3 Inflammasomes Formation via SIRT1-NRF2 Signaling. ACS Omega.

[70] Laavola M., Nieminen R., Yam M., Sadikun A., Asmawi M., Basir R., Welling J., Vapaatalo H., Korhonen R., Moilanen E. (2012). Flavonoids Eupatorin and Sinensetin Present in *Orthosiphon stamineus* Leaves Inhibit Inflammatory Gene Expression and STAT1 Activation. Planta Med..

[71] Wang Y.-L., Yang J.-J., Ni W. (2022). Immunomodulatory Effects of Sinensetin on Macrophage and Cyclophosphamide-Induced Immunosuppression in Mice. Die Pharm..

[72] Xu Z., Hu H., Wang K., Zhou Z., He X., Huang X., Hu Y., Huang J., Luo Z. (2024). Sinensetin, a Polymethoxyflavone from Citrus Fruits, Ameliorates LPS-Induced Acute Lung Injury by Suppressing Txnip/NLRP3/Caspase-1/GSDMD Signaling-Mediated Inflammatory Responses and Pyroptosis. Food Funct..

[73] Jiang X., Shon K., Li X., Cui G., Wu Y., Wei Z., Wang A., Li X., Lu Y. (2024). Recent Advances in Identifying Protein Targets of Bioactive Natural Products. Heliyon.

[74] Tabana Y., Babu D., Fahlman R., Siraki A.G., Barakat K. (2023). Target Identification of Small Molecules: An Overview of the Current Applications in Drug Discovery. BMC Biotechnol..

[75] Ha J., Park H., Park J., Park S.B. (2021). Recent Advances in Identifying Protein Targets in Drug Discovery. Cell Chem. Biol..

[76] Yoshida M. (2018). Recent Advances in Target Identification of Bioactive Natural Products. Biosci. Biotechnol. Biochem..

[77] Li G., Peng X., Guo Y., Gong S., Cao S., Qiu F. (2021). Currently Available Strategies for Target Identification of Bioactive Natural Products. Front. Chem..

[78] Lipinski C.A., Lombardo F., Dominy B.W., Feeney P.J. (1997). Experimental and Computational Approaches to Estimate Solubility and Permeability in Drug Discovery and Development Settings. Adv. Drug Deliv. Rev..

[79] Hossainab M.A., Ismail Z. (2004). Synthesis of Sinensetin, A Naturally Occurring Polymethoxyflavone. Pak. J. Sci. Ind. Res..

[80] Paggi J.M., Pandit A., Dror R.O. (2024). The Art and Science of Molecular Docking. Annu. Rev. Biochem..

[81] He C., Hao E., Du C., Wei W., Wang X., Liu T., Deng J. (2023). Investigating the Underlying Mechanisms of Ardisia Japonica Extract’s Anti-Blood-Stasis Effect via Metabolomics and Network Pharmacology. Molecules.

[82] Li X., Wang Q., Wang M., Liu Y., Chen L., Wang F., Chen H. (2024). Integrated Metabolomics and Network Pharmacology Revealed the Key Active Ingredients for the Treatment of Ulcerative Colitis in the Citrus Reticulata ‘Dahongpao’ Peel. J. Pharm. Biomed. Anal..

[83] Kori M., Temiz K., Gov E. (2023). Network Medicine Approaches for Identification of Novel Prognostic Systems Biomarkers and Drug Candidates for Papillary Thyroid Carcinoma. J. Cell. Mol. Med..

[84] Yuan Z., Li J., Xiao F., Wu Y., Zhang Z., Shi J., Qian J., Wu X., Yan F. (2024). Sinensetin Protects against Periodontitis Through Binding to Bach1 Enhancing Its Ubiquitination Degradation and Improving Oxidative Stress. Int. J. Oral Sci..

[85] Liu D., Cao X., Kong Y., Mu T., Liu J. (2021). Inhibitory Mechanism of Sinensetin on α-Glucosidase and Non-Enzymatic Glycation: Insights from Spectroscopy and Molecular Docking Analyses. Int. J. Biol. Macromol..

[86] Rajan P., Natraj P., Ranaweera S.S., Dayarathne L.A., Lee Y.J., Han C.-H. (2021). Anti-Adipogenic Effect of the Flavonoids through the Activation of AMPK in Palmitate (PA)-Treated HepG2 Cells. J. Vet. Sci..

[87] Androutsopoulos V.P., Ruparelia K., Arroo R.R.J., Tsatsakis A.M., Spandidos D.A. (2009). CYP1-Mediated Antiproliferative Activity of Dietary Flavonoids in MDA-MB-468 Breast Cancer Cells. Toxicology.

[88] Pan Y., Tiong K.H., Abd-Rashid B.A., Ismail Z., Ismail R., Mak J.W., Ong C.E. (2014). In Vitro Effect of Important Herbal Active Constituents on Human Cytochrome P450 1A2 (CYP1A2) Activity. Phytomedicine.

[89] Bai J., Li L., Zhao S., Fan X., Zhang J., Hu M., Chen Y., Sun Y., Wang B., Jin J. (2020). Heterotropic Activation of Flavonoids on Cytochrome P450 3A4: A Case Example of Alleviating Dronedarone-Induced Cytotoxicity. Toxicol. Lett..

[90] Feng F., Li T., Liang Y., Gao W., Yang L. (2023). Structural Changes and Anti-Hepatocellular Carcinoma Activity of Interferon-γ after Interaction with Sinensetin. Int. J. Biol. Macromol..

[91] Zhang L., Zheng J., Ma M., Zhao Y., Song J., Chen X., Cao W., He X., Xue C., Tang Q. (2021). Drug-Guided Screening for Pancreatic Lipase Inhibitors in Functional Foods. Food Funct..

[92] Malterud K.E., Rydland K.M. (2000). Inhibitors of 15-Lipoxygenase from Orange Peel. J. Agric. Food Chem..

[93] Duan X., Bai W., Hu J., Wu J., Tan H., Wang F., Lang X., Wang B., Hu J. (2024). Inhibitory Effect of Flavonoids on Multidrug and Toxin Extrusion Protein 1 Function: Implications for Food/Herb–Drug Interaction and Drug-induced Kidney Injury. J. Appl. Toxicol..

[94] Fan X., Bai J., Hu M., Xu Y., Zhao S., Sun Y., Wang B., Hu J., Li Y. (2020). Drug Interaction Study of Flavonoids toward OATP1B1 and Their 3D Structure Activity Relationship Analysis for Predicting Hepatoprotective Effects. Toxicology.

[95] Bajraktari-Sylejmani G., Weiss J. (2020). Potential Risk of Food-Drug Interactions: Citrus Polymethoxyflavones and Flavanones as Inhibitors of the Organic Anion Transporting Polypeptides (OATP) 1B1, 1B3, and 2B1. Eur. J. Drug Metab. Pharmacokinet..

[96] Hubbard B.P., Gomes A.P., Dai H., Li J., Case A.W., Considine T., Riera T.V., Lee J.E., E S.Y., Lamming D.W. (2013). Evidence for a Common Mechanism of SIRT1 Regulation by Allosteric Activators. Science.

[97] Lakshminarasimhan M., Rauh D., Schutkowski M., Steegborn C. (2013). Sirt1 Activation by Resveratrol Is Substrate Sequence-Selective. Aging.

[98] Yang Z.-Y., Dong J., Yang Z.-J., Lu A.-P., Hou T.-J., Cao D.-S. (2020). Structural Analysis and Identification of False Positive Hits in Luciferase-Based Assays. J. Chem. Inf. Model..

